# Evaluation of venous thromboembolism and malignancy development in splenectomized patients: A retrospective single-center study from a Southern Anatolian City (Isparta)

**DOI:** 10.1097/MD.0000000000047667

**Published:** 2026-02-13

**Authors:** Emre Karanfil, Alparslan Merdin, Elif Selin Karanfil

**Affiliations:** a Department of Internal Medicine, Süleyman Demirel University Faculty of Medicine, Isparta, Turkey; b Division of Hematology, Department of Internal Medicine, Süleyman Demirel University Faculty of Medicine, Isparta, Turkey; c Department of Physiology, Süleyman Demirel University Faculty of Medicine, Isparta, Turkey.

**Keywords:** malignancy, platelet, splenectomy, thrombosis

## Abstract

This study investigates the incidence, anatomical localization, and time to onset of deep vein thrombosis and venous thromboembolism (VTE) following splenectomy. A secondary objective was to identify which malignancies developed after splenectomy, as well as the time interval between splenectomy and the detection of these malignancies. We retrospectively analyzed 189 patients who underwent splenectomy at Süleyman Demirel University Research and Training Hospital between January 01, 2005 and July 07, 2023. Post-procedural VTE and malignancy development, splenectomy etiologies, and serial platelet counts were evaluated. Patients were stratified for subgroup analysis. Higher pre-splenectomy platelet counts are associated with an increased risk of early post-procedural thrombosis. Platelet levels do not appear to influence the long-term risk of malignancy.

**Results:**

A significant association was found between the last pre-splenectomy platelet count and the development of thrombosis within the first postoperative month (*P* = .045). Overall, VTE occurred in 4.2% of patients within the first month, with portal vein thrombosis being the most common type in both early and late periods. Persistent thrombocytosis (platelets > 4,50,000/mm^3^) was present in 29.3% of patients at 1 to 6 months and 10.9% after 6 months. In the immune thrombocytopenic purpura subgroup, 71.2% of patients maintained a platelet count above 30,000/mm^3^ after 6 months. We found no significant association between pre- or post-splenectomy platelet levels and the subsequent development of malignancy.

## 1. Introduction

Splenectomy serves a dual role in clinical practice as a life-saving procedure following trauma and as a therapeutic intervention for several hematological disorders. It remains a cornerstone of treatment for conditions such as thalassemia major, immune thrombocytopenia/idiopathic thrombocytopenic purpura (ITP), and autoimmune hemolytic anemia.

Given the spleen’s integral role in the immune system, its removal raises important clinical questions regarding long-term patient outcomes. The potential for splenectomy to increase the risk of subsequent malignancy and venous thromboembolism (VTE) has become a significant area of investigation. While post-splenectomy management involves specific vaccination protocols to mitigate infectious risks, the commonly observed reactive thrombocytosis presents a distinct clinical challenge. Unlike primary clonal thrombocytosis (e.g., essential thrombocytosis), which carries a recognized high risk for thrombosis, the reactive thrombocytosis following splenectomy is not typically managed with routine antithrombotic prophylaxis.

A Danish study by Thomsen et al, for example, reported a VTE incidence of 1.86% within the first 90 days post-splenectomy, a rate substantially higher than that observed in the general population.^[1]^ Corroborating these findings, a large study of 8149 patients by Kristinsson et al demonstrated that splenectomy is associated with an increased risk of both deep vein thrombosis (DVT) and pulmonary embolism.^[2]^ Their research also revealed a heightened risk for various solid organ cancers – including those of the esophagus, colon, lung, and liver – and several hematological malignancies, such as non-Hodgkin lymphoma, Hodgkin lymphoma, and various forms of leukemia.^[2]^

The aim of this study was to determine the incidence of DVT or VTE after splenectomy, the specific veins in which DVT/VTE developed, and the time interval between splenectomy and the occurrence of these events. Another objective was to identify which malignancies developed after splenectomy, as well as the time interval between splenectomy and the detection of these malignancies. Investigating thrombocytosis levels in patients following splenectomy was also among the study objectives. Finally, we characterized post-procedural platelet dynamics and investigated whether early thrombocytosis, specifically platelet counts within the first month post-surgery, was associated with the development of thromboembolic events.

## 2. Materials and methods

### 2.1. Study population and design

This retrospective study involved the review of medical records for 189 patients who underwent splenectomy at Süleyman Demirel University Faculty of Medicine Hospital between January 01, 2005 and July 07, 2023. The study cohort was restricted to individuals over 18 years of age at the time of their procedure. We collected and analyzed the demographic characteristics of all included patients.

### 2.2. Exclusion criteria

We established several exclusion criteria for the primary analysis. Patients were excluded if they had a known malignancy prior to splenectomy (except for basal cell carcinoma of the skin) or if a malignancy was diagnosed concurrently from the surgical pathology. We also excluded individuals with a documented history of several preexisting conditions, including organ or stem cell transplantation, aplastic anemia, myeloproliferative disorders, Fanconi syndrome, and congenital immunodeficiency. Additional exclusion criteria were a history of inflammatory bowel disease (ulcerative colitis or Crohn’s disease), preexisting liver cirrhosis, documented human immunodeficiency virus infection, or the use of non-steroidal immunosuppressive therapy for inflammatory connective tissue diseases.

### 2.3. Data collection

For each patient in the cohort, we extracted data from hospital archives and medical records. The clinical indications for splenectomy (e.g., trauma, ITP, autoimmune hemolytic anemia, diagnostic purposes) were identified and tabulated. Longitudinal platelet count data, including the last measurement prior to splenectomy, the highest and lowest values within the first postoperative month, the lowest value between 1 and 6 months, and the lowest value beyond 6 months post-procedure were collected, and from these data, both mean and median values for each time point were calculated.

### 2.4. Primary analyses

The study was designed to investigate 2 primary outcomes: post-splenectomy malignancy and VTE. We first analyzed the incidence and characteristics of malignancies that developed following splenectomy, which involved identifying the specific cancer types and determining the time interval between the surgical procedure and the subsequent diagnosis. For this outcome, we applied additional exclusion criteria, removing patients from the analysis who had a history of chronic hepatitis B or C virus infection, known smoldering myeloma prior to splenectomy, or a genetically confirmed diagnosis of hemochromatosis. Patients who were lost to follow-up at our institution after their initial discharge were also excluded from this specific analysis.

Our second primary analysis focused on the incidence and characteristics of post-splenectomy VTE, including DVT. For patients who experienced a thrombotic event, we identified the affected anatomical veins and determined the duration from surgery to the event. A separate set of exclusion criteria was applied to this cohort. We excluded patients with preexisting conditions known to confer a high thrombotic risk, such as a history of vasculitis, Behçet’s disease, hereditary thrombophilia, paroxysmal nocturnal hemoglobinuria, or prior DVT/VTE. Additional exclusions comprised individuals with hemiplegia or tetraplegia, patients with hemophilia receiving prophylactic factor replacement, and those on long-term anticoagulation for non-thrombotic cardiac reasons (e.g., atrial fibrillation, prosthetic heart valves). Finally, we excluded patients whose sole thrombotic event was documented during an active COVID-19 infection.

### 2.5. Subgroup analysis

A specific subgroup analysis was conducted to explore the relationship between early postoperative thrombocytosis and VTE. Within the thromboembolism cohort, patients whose indication for splenectomy was ITP were first excluded. In the remaining patients, we then assessed the association between platelet counts (both highest and lowest values) within the first month and the development of early-onset DVT/VTE, defined as occurring within 30 days of the procedure. To facilitate this analysis, patients were stratified based on whether they developed a thrombotic event within this early period.

### 2.6. Ethical considerations and data management

This retrospective study was conducted in accordance with the ethical principles outlined in the Declaration of Helsinki. The research protocol was reviewed and approved by the Süleyman Demirel University Faculty of Medicine Clinical Research Ethics Committee on July 21, 2023 (Decision No. 152). All patient data were extracted from the hospital’s electronic medical record system and archived files. To ensure confidentiality, the dataset was fully anonymized prior to analysis, and no personally identifiable information was used or publicly shared. The final date for patient follow-up was defined as their last recorded hospital visit before March 10, 2025.

### 2.7. Statistical analysis

Descriptive data were presented as follows: categorical variables grouped by frequency (n) and percentages, while numerical (measurement) data were presented as mean ± standard deviation, median with 25th to 75th percentile ranges, and minimum–maximum values. Tables and graphs were utilized for data presentation.

Prior to conducting analyses, normality distribution of general numerical data and numerical data to be compared between groups was assessed using Kolmogorov–Smirnov and Shapiro–Wilk tests. Normality assessment of numerical data was supported by histogram graph evaluation. For numerical variables following normal distribution, mean ± standard deviation and parametric tests were used, while for variables not following normal distribution, median, 25th to 75th percentile ranges, and non-parametric tests were employed.

For numerical variables that did not follow normal distribution according to Kolmogorov-Smirnov and Shapiro–Wilk tests, skewness and kurtosis values were additionally examined. Numerical variables with values between −1.5 and +1.5 were considered to follow normal distribution and were presented as mean ± standard deviation, with parametric tests applied. For numerical data with sample sizes >50, Kolmogorov–Smirnov test results were considered, while for sample sizes of 50 or fewer, Shapiro–Wilk test results were used.

All collected data were entered into Statistical Package for the Social Sciences (SPSS) version 22 (IBM Corp., Armonk) software package and analyzed through the same program. For comparisons of continuous data, parametric tests were used for normally distributed variables and non-parametric tests for those that were not. To compare independent groups, the independent samples *t*-test (parametric) or the Mann–Whitney *U* and Kruskal–Wallis tests (non-parametric) were employed. For dependent (paired) groups, the paired samples *t*-test (parametric) or the Wilcoxon and Friedman tests (non-parametric) were used.

Correlation between continuous variables was analyzed via the Pearson correlation test for normally distributed data and the Spearman’s correlation test for non-normally distributed data. For the comparison of independent categorical data, the Pearson chi-square, Yates’ continuity correction, and Fisher’s exact tests were used. For dependent categorical data, the McNemar test was applied. For all statistical tests, a 2-tailed *P*-value < .05 was considered statistically significant.

## 3. Results

### 3.1. Patient demographics and splenectomy etiology

The study cohort consisted of 189 patients, of whom 102 (54.0%) were male. The clinical indications for splenectomy were determined for 187 of these patients. The most frequent etiology was trauma-related splenic laceration or rupture, accounting for 40.1% (n = 75) of cases, followed by ITP at 16.6% (n = 31).

A variety of other clinical indications were identified. Cystic lesions were a common reason, including hydatid cysts (7.5%, n = 14) and other benign cystic lesions (9.6%, n = 18). Other notable etiologies included thalassemia (5.3%, n = 10), splenic abscess (3.2%, n = 6), ileus (3.2%, n = 6), splenic infarction (2.1%, n = 4), hemolytic anemia (1.6%, n = 3), hypersplenism (1.6%, n = 3), aortic aneurysm (1.1%, n = 2), gastrointestinal bleeding (1.1%, n = 2), and hereditary spherocytosis (1.1%, n = 2). Finally, a number of rare conditions, each accounting for a single case (0.5%), were confirmed as the indication for splenectomy: granulomatous infection, splenic hamartoma, diaphragmatic hernia, granulomatous pyelonephritis, hemangioma, hiatal hernia, lymphangioma, mucinous cystadenoma, necrotizing pancreatitis, other hemoglobinopathies, and fibroadipose tissue of the pancreas. The indication for the procedure could not be determined from the available records for the remaining 2 patients, a limitation inherent to the study’s retrospective design.

### 3.2. Post-splenectomy malignancy and thrombosis

The types of malignancies developing post-splenectomy and the interval to their development are detailed in Table 1. The anatomical locations of all venous thrombosis events are presented in Table 2.

**Table 1:** Distribution of post-splenectomy malignancy types and time to development.

Malignancy type	n	%	Time to development (mo)
Colon cancer	2	25	3–14
Lung cancer	1	12.5	97
Follicular thyroid cancer	1	12.5	75
Hepatocellular carcinoma	1	12.5	88
Breast cancer	1	12.5	158
Pancreatic cancer	1	12.5	227
Papillary thyroid cancer	1	12.5	37
Total	8	100	

**Table 2:** Anatomical distribution of post-splenectomy venous thrombosis.

Venous thrombosis location	n	%
Portal vein thrombosis	14	58.3
Deep vein thrombosis, other	3	12.5
Pulmonary embolism	2	8.3
Basilic vein thrombosis	1	4.2
Internal jugular vein thrombosis	1	4.2
Superior sagittal sinus thrombosis	1	4.2
Left common and internal iliac thrombosis	1	4.2
Pulmonary embolism with deep vein thrombosis	1	4.2
Total	24	100

Overall, post-splenectomy VTE, DVT, or portal vein thrombosis was identified in 12.7% (n = 24) of patients. A thrombotic event occurred within the first month of the procedure in 4.2% (n = 8) of the total patient cohort. The median time to the development of venous thrombosis post-splenectomy was 5.5 months (range: 1–24 months). The distribution of venous thrombosis types according to the time of onset after splenectomy is shown in Table 3.

**Table 3:** Distribution of venous thrombosis type by time of onset post-splenectomy.

Thrombosis type	<1 mo	1–6 mo	>6 mo
Portal vein thrombosis	6	3	5
Deep vein thrombosis, other	–	2	1
Pulmonary embolism	1	–	1
Basilic vein thrombosis	–	1	–
Internal jugular vein thrombosis	–	–	1
Superior sagittal sinus thrombosis	–	–	1
Left common and internal iliac thrombosis	1	–	–
Pulmonary embolism with DVT	–	–	1
Total n (%)	8 (33.3)	6 (25.0)	10 (41.7)

The values in the table represent the number of events (n). En dashes (–) indicate 0 events in a category.

The percentages in the total row are based on the total of 24 thrombotic events.

DVT = deep vein thrombosis.

We observed significant gender-based differences related to the primary indications for splenectomy. In cases necessitated by trauma, the proportion of male patients was significantly higher than that of females (47.5% vs 31.4%; χ^2^ (1) = 5.031, *P* = .025). Conversely, for splenectomies performed for ITP, female patients constituted a significantly larger proportion than males (27.9% vs 6.9%; χ^2^ (1) = 13.301, *P* < .001). The longitudinal platelet counts for the trauma subgroup are detailed in Table 4.

**Table 4:** Pre- and post-splenectomy platelet levels in patients splenectomized for trauma (n = 75).

Platelet measurement	n	Mean ± SD (×10^3^/mm^3^)
Pre-splenectomy (last available)	72	228.0 ± 86.9
Post-splenectomy peak (1st mo)	59	644.8 ± 366.1
Post-splenectomy nadir (1–6 mo)	22	394.6 ± 151.1
Post-splenectomy nadir (>6 mo)	22	307.8 ± 148.3

The column n represents the number of patients with available data for each measurement.

SD = standard deviation.

We next investigated whether platelet levels were associated with the long-term risk of malignancy. A comparison of platelet counts at various pre- and postoperative time points, presented in Table 5, showed no statistically significant differences between patients who subsequently developed malignancy and those who did not (*P* > .05 for all comparisons).

**Table 5:** Comparison of platelet levels based on post-splenectomy malignancy development.

Platelet measurement	Malignancy yes: n	Malignancy: median (IQR)	Malignancy no: n	No malignancy: median (IQR)	*P*
Pre-splenectomy (last available)	8	208 (166.5–296.5)	177	203 (134–278)	.62
Post-splenectomy nadir (1st mo)	8	184.5 (134–337)	160	164.5 (103.5–226)	.294
Post-splenectomy peak (1st mo)	8	611.5 (401.5–741)	160	606.5 (383–800)	.994
Post-splenectomy nadir (1–6 mo)	6	301 (183–404)	86	358 (256–466)	.389
Post-splenectomy nadir (>6 mo)	7	254 (178–296.5)	94	283.5 (165–355)	.597

Platelet levels are presented in ×10^3^/mm^3^.

*P*-values were calculated using the Mann–Whitney *U* test.

The column *n* represents the number of patients with available data for each measurement.

IQR = interquartile range.

**Figure 1.**
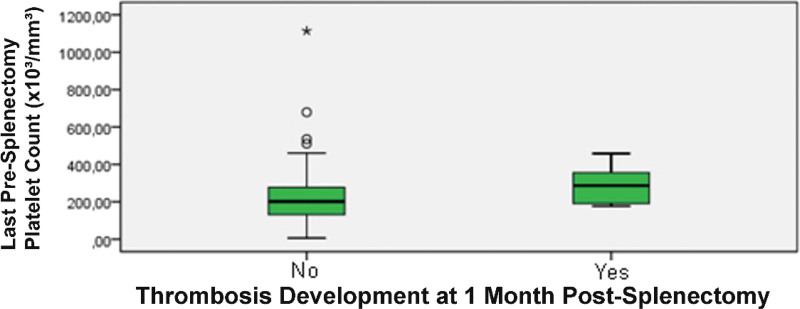
Comparison of pre-splenectomy platelet counts and early venous thrombosis. The boxplot displays the distribution of the last pre-splenectomy platelet count in patients who did and did not develop venous thrombosis within the first postoperative month. The difference between the groups is statistically significant (*P* = .045, Mann–Whitney *U* test).

In contrast to the malignancy analysis, we identified a significant predictor for early postoperative venous thrombosis. As illustrated in Figure 1, patients who developed a thrombotic event within the first month had a significantly higher median pre-splenectomy platelet count than those who did not (287 × 10^3^/mm^3^ [IQR: 191–355.5] vs 201 × 10^3^/mm^3^ [IQR: 131–277]; *P* = .045).

The association appeared specific to the pre-operative platelet count and the early postoperative period. We found no significant relationship between the underlying etiology of the splenectomy and the risk of developing VTE within the first 6 months (*P* > .05). Besides, in a sub-analysis excluding patients with ITP, neither the peak nor the nadir platelet counts during the first month were associated with early thrombosis (*P* > .05). The overall impact of splenectomy on platelet counts was then assessed for the entire cohort, as detailed in Table 6.

**Table 6:** Paired comparison of pre-splenectomy and peak post-splenectomy platelet levels.

Time point	n	Median (IQR; ×10^3^/mm^3^)
Pre-splenectomy (last available)	185	203 (135.5–282.5)
Post-splenectomy peak (1st mo)	168	606.5 (388.8–796.8)

The statistical comparison between the 2 time points was performed on paired data via the Wilcoxon signed-rank test, which yielded a significant difference (*P* < .001).

IQR = interquartile range.

### 3.3. Temporal trends in platelet counts

The median peak platelet count within the first month was ~3 times higher than the median pre-splenectomy baseline (606.5 vs 203 × 10^3^/mm^3^; *P* < .001), as detailed in Table 6. This thrombocytosis appeared to diminish over time. A paired-samples analysis showed that the mean nadir platelet count was significantly lower at >6 months post-procedure compared to the 1 to 6-month interval, indicating a gradual downward trend (Table 7).

**Table 7:** Paired comparison of nadir platelet levels at 1 to 6 months and >6 months post-splenectomy.

Measurement	1–6 mo post-splenectomy	>6 mo post-splenectomy	*P*
Mean ± SD	335.1 ± 190.2	273.6 ± 173.3	<.001

n = 80.

Platelet levels are presented in ×10^3^/mm^3^.

The *P*-value was calculated via a paired-samples *t*-test.

SD = standard deviation.

The mean platelet levels measured >6 months post-splenectomy (273.6 ± 173.3 × 10^3^/mm^3^) were found to be lower as compared to the mean platelet levels measured between 1 to 6 months post-splenectomy (335.1 ± 190.2 × 10^3^/mm^3^), which was statistically significant (*P* < .001).

## 4. Discussion

Splenectomy has been a critical therapeutic intervention for select hematological disorders and a necessary procedure following severe abdominal trauma. Consistent with previous reports, such as those by Erten et al,^[3]^ the most common indication for splenectomy in our cohort was trauma, followed by ITP. This confirms that traumatic splenic injury remains a primary driver of spleen loss in the general population, reinforcing the clinical importance of understanding the long-term sequelae in this large patient group.

Our analysis of post-splenectomy platelet dynamics revealed a significant, albeit waning, trend of thrombocytosis. While nearly 70% of patients exhibited thrombocytosis (platelets > 450 × 10^3^/mm^3^) within the first month, this proportion decreased to ~11% after 6 months. This temporal pattern underscores that while reactive thrombocytosis is a hallmark of the early post-splenectomy period, a persistent elevation in platelet count can remain a long-term clinical consideration and should be recognized as a potential secondary cause in the differential diagnosis of thrombocytosis.

A key finding of our study is the association between pre-splenectomy platelet count and the risk of early postoperative VTE. Patients who developed thrombosis within the first month had a significantly higher median pre-operative platelet count compared to those who did not (287 vs 201 × 10^3^/mm^3^). This suggests that the baseline platelet level may be a valuable biomarker for stratifying patients at high risk for immediate thrombotic complications. However, prospective studies with larger cohorts are needed to validate this association and to determine a specific platelet threshold at which this risk becomes clinically significant.

Schilling et al demonstrated a markedly increased long-term risk for both arterial (hazard ratio 7.2) and venous (hazard ratio 3.3) events in splenectomized patients with hereditary spherocytosis.^[4]^ Likewise, Capellini et al reported a high rate of thromboembolic events (~29%) in patients with thalassemia intermedia who had undergone splenectomy.^[5]^ In our more heterogeneous cohort, the overall incidence of postoperative thrombosis was 12.7% (n = 24), with a median time to onset of 5.5 months. Critically, one-third of these events, representing 4.2% of all splenectomized patients in our study, occurred within the first 30 days, which highlights the early postoperative period as a time of peak vulnerability. Consistent with this observation, Faucher et al found that thromboembolic events occurred in 6.7% of splenectomized onco-hematologic patients within the first 90 days, increasing to 11.7% at 1 year, underscoring the substantial thrombotic burden in this population.^[6]^ Moreover, Baldari et al reported porto-spleno-mesenteric venous thrombosis in 6 of 22 patients (27.3%) after elective splenectomy, further emphasizing the procedure’s inherent predisposition to venous thrombotic complications.^[7]^ Taken together, these findings highlight the need for heightened vigilance for VTE, particularly during the early postoperative phase. Given the temporal clustering of events in the high-risk early period, our results support considering anticoagulant prophylaxis during this high-risk period, especially for patients with additional risk factors.

Our analysis of thrombosis localization showed that events occurred most frequently in the portal and splanchnic venous systems. This finding is consistent with the literature, including a report by Abduljalil et al which identified the same region as the most common site for post-splenectomy thrombosis.^[8]^ Regarding timing, we observed that portal vein thrombosis was a risk in both the early and late postoperative periods, developing within the first month in some cases and after 6 months in others. This highlights the need for sustained clinical vigilance. Nevertheless, our findings reinforce that patients require careful monitoring for splanchnic venous thrombosis not only immediately after surgery but also during long-term follow-up.

We also evaluated the long-term efficacy of splenectomy for patients with ITP. In our cohort, 71.2% of patients maintained a platelet count of <30,000/mm^3^ beyond 6 months, a rate we considered a durable long-term response. For comparison, a study by Schwartz et al used more stringent criteria, defining a “complete response” as a platelet count > 150 × 10^9^/L and a “partial response” as ≥50 × 10^9^/L, both without treatment.^[9]^ They reported that 57% of their patients maintained a complete response long-term.^[9]^ Although our study’s response threshold was lower, our findings align with those of Schwartz et al in demonstrating that splenectomy is an effective and durable treatment for a substantial majority of patients with refractory ITP.

Our findings on post-splenectomy malignancy differ from some previous reports. Linet et al, for instance, identified breast cancer as the most common malignancy in a cohort of patients splenectomized for trauma.^[10]^ In our study, however, colon cancer was the most frequently observed type. It is also important to consider the findings of Thai et al, who reported that malignancy incidence was comparable between ITP patients who did and did not undergo splenectomy.^[11]^ In our long-term follow-up, we detected a range of solid organ tumors, including colon, lung, thyroid, hepatocellular, breast, and pancreatic cancers. Our analysis revealed no significant association between pre- or post-splenectomy platelet levels and the subsequent development of malignancy, suggesting that platelet count is not a primary driver of this risk.

### 4.1. Study limitations

This study has several limitations inherent to its retrospective design. First, we could not evaluate comorbid conditions that may contribute to thrombosis, such as smoking, obesity, or the use of oral contraceptives. Similarly, potential risk factors for malignancy, including smoking, chronic constipation, or family history, could not be assessed. Consequently, the analyses relied solely on the data available in our patient records, which may be incomplete.

## 5. Conclusion

In conclusion, our study affirms several key clinical considerations for the long-term management of splenectomized patients. Clinicians should remain mindful of the potential for malignancy, and these patients should continue to follow standard cancer screening guidelines. Furthermore, the risk of thrombosis, particularly portal vein thrombosis in the early postoperative period, necessitates careful and vigilant patient follow-up. For patients with relapsed/refractory ITP, splenectomy remains an effective option to be considered when clinically indicated. Future prospective studies comparing splenectomized and non-splenectomized patients within more homogeneous, well-defined cohorts are needed to draw more definitive conclusions regarding these long-term risks.

## Acknowledgments

The authors wish to thank Erman Zengin, MD, for his assistance with the statistical analysis. This manuscript was derived from the internal medicine specialty thesis of Emre Karanfil, completed in August 2025 at Süleyman Demirel University Faculty of Medicine, Department of Internal Medicine, Isparta, Turkey. Alparslan Merdin is the thesis advisor.

## Author contributions

**Conceptualization:** Alparslan Merdin.

**Data curation:** Emre Karanfil.

**Formal analysis:** Emre Karanfil, Elif Selin Karanfil.

**Investigation:** Emre Karanfil, Elif Selin Karanfil.

**Methodology:** Alparslan Merdin.

**Supervision:** Alparslan Merdin.

**Writing – review & editing:** Emre Karanfil, Elif Selin Karanfil.
